# Measurement and monitoring of safety: impact and challenges of putting a conceptual framework into practice

**DOI:** 10.1136/bmjqs-2017-007175

**Published:** 2018-03-06

**Authors:** Eleanor Chatburn, Carl Macrae, Jane Carthey, Charles Vincent

**Affiliations:** 1 Department of Experimental Psychology, University of Oxford, Oxford, UK; 2 Jane Carthey Consulting, London, UK

**Keywords:** patient safety, qualitative research, safety culture, quality measurement

## Abstract

**Background:**

The Measurement and Monitoring of Safety Framework provides a conceptual model to guide organisations in assessing safety. The Health Foundation funded a large-scale programme to assess the value and impact of applying the Framework in regional and frontline care settings. We explored the experiences and reflections of key participants in the programme.

**Methods:**

The study was conducted in the nine healthcare organisations in England and Scotland testing the Framework (three regional improvement bodies, six frontline settings). Post hoc interviews with clinical and managerial staff were analysed using template analysis.

**Findings:**

Participants reported that the Framework promoted a substantial shift in their thinking about how safety is actively managed in their environment. It provided a common language, facilitated a more inquisitive approach and encouraged a more holistic view of the components of safety. These changes in conceptual understanding, however, did not always translate into broader changes in practice, with many sites only addressing some aspects of the Framework. One of the three regions did embrace the Framework in its entirety and achieved wider impact with a range of interventions. This region had committed leaders who took time to fully understand the concepts, who maintained a flexible approach to exploring the utility of the Framework and who worked with frontline staff to translate the concepts for local settings.

**Conclusions:**

The Measuring and Monitoring of Safety Framework has the potential to support a broader and richer approach to organisational safety. Such a conceptually based initiative requires both committed leaders who themselves understand the concepts and more time to establish understanding and aims than might be needed in a standard improvement programme.

## Background

The measurement and monitoring of safety continues to be a priority for all healthcare systems. While the extent of serious harm from healthcare, and in particular the mortality from unsafe care, is much debated, there is little doubt that care is often unreliable and sometimes harmful.^1–3^ To make healthcare safer, organisations need to continually measure harm and reliability to assess standards of care and target programmes of improvement.^4 5^ They also need to remain alert to problems and perturbation as they occur, and be adept at responding to and managing potential threats to safety.^6–9^

The nature of safety has been discussed in the wider literature but, in healthcare at least, there has been little consensus on the core dimensions of safety or what exactly should be measured and monitored.^10–12^ While many authors have suggested that more attention should be given to proactive approaches to assessing the safety capacity of organisations, much less attention has been given to how this might work in practice.^13–15^ We previously published a report which sought to capture and integrate all the critical dimensions of safety in one framework and provided examples of how these various concepts might be realised in practice within organisations. The Measurement and Monitoring of Safety (MMS) Framework attempted to synthesise the wider theory, literature and practice from both healthcare and other industries in a form which aimed to be accessible and useful to healthcare organisations (box 1). Rather than being led by available data, clinicians, managers and board members could use the Framework to consider what information they really needed and how they might develop a more nuanced and comprehensive approach.^10^

Early discussion and preliminary testing suggested the MMS Framework was useful in providing structure and clarity to discussions about safety, allowing staff at pilot sites to better organise and understand their existing measures.^16^ It also promoted a realisation that their current ability to reflect on their organisation’s safety was almost entirely reliant on analyses of past harm.^17 18^ This initial testing indicated that the Framework, and the attitude of inquiry that it embodied, could play a part in a more fundamental shift: from an unthinking reliance on regulatory compliance as the guarantor of safety—a mindset of assurance—to a more proactive approach of intelligent measurement and monitoring— a mindset of inquiry. Following these pilot study findings,^16^ The Health Foundation commissioned the MMS Programme, a major initiative intended to test how the Framework might be applied and adapted in a variety of healthcare settings (box 1).

In this paper we report on the experiences of the MMS Programme participants who tested the MMS Framework. The present study sought to address the following questions: (1) What was the role of the Framework in the MMS Programme and how was the Framework understood? (2) What was the impact of the Framework in the participating test site organisations? (3) What are the implications for the wider use and application of this new approach to measuring and monitoring safety?

Box 1 **The measuring and monitoring of safety**

**The Measuring and Monitoring of Safety Framework**

The Measurement and Monitoring of Safety (MMS) Framework encompasses the five principal dimensions of safety that enable an organisation to assess whether care is safe:

- Has patient care been safe in the past? We need to assess rates of past harm to patients, both physical and psychological.
- Are our clinical systems and processes reliable? This is the reliability of safety-critical processes and systems but also the capacity of the staff to follow safety-critical procedures.
- Is care safe today? This is the information and capacity to monitor safety on an hourly or daily basis. We refer to this as ‘sensitivity to operations’.
- Will care be safe in the future? This refers to the ability to anticipate, and be prepared for, problems and threats to safety.
- Are we responding and improving? The capacity of an organisation to detect, analyse, integrate, respond and improve from safety information.

**The Measurement and Monitoring of Safety Programme**

The objective of the MMS Programme, as specified by The Health Foundation, was to test and further develop the Framework in a variety of National Health Service (NHS) organisations. The nature of the testing was not closely specified but can be broadly understood as exploring and assessing the utility and impact of the Framework and associated materials in NHS organisations. The programme ran from January 2015 to June 2016. Three regional improvement bodies in England and Scotland successfully bid to participate, each nominating two frontline NHS organisations within their regions.

Substantial funding was allocated to the organisation of the programme and to the nine organisations. Regional improvement bodies had a senior lead and a full-time programme manager for the duration of the programme. The core programme team at each frontline organisation consisted of: a full-time project lead, a manager responsible for quality and safety assurance, and one or more lead clinicians from their nominated test sites. An external provider facilitated four national programme learning events, which provided support and opportunities to share learning, with some input from the Framework experts.

Participating organisations were given the freedom to decide how they wished to apply the Framework within their local context, with the primary focus being translation to frontline practice in a small number of test wards or units. Examples of themes to this work included: multidisciplinary team communication, medications safety, mapping patient pathways, and predicting and preventing incidents of violence and aggression.^21^ Sites were encouraged to capture their learning on the suitability of applying the Framework in their settings and to adapt it freely as required.

## Methods

We carried out semistructured interviews with senior and frontline participants in the MMS Programme. The interviews explored how the Framework had been used and applied, how the Framework had influenced people’s understanding and knowledge of safety, how people believed the Framework had impacted organisational practice, and the perceived potential for wider application of the Framework. This analysis was supported and informed by documentary review of programme materials produced by participating sites over 18 months.

### Settings

Three regional improvement bodies in England and Scotland led the programmes within their region, each working with two frontline National Health Service organisations. These sites consisted of two combined health boards, two mental health trusts, one acute trust and one ambulance trust. There were significant variations in service delivery, locations, organisational structures and progress on the safety agenda across these test sites.

### Participants and interviews

Interviews were conducted with 28 MMS Programme participants across nine research sites. Participants were selected through purposive sampling to identify individuals at each research site working in each of the key roles on the programme. One senior director and one programme manager were interviewed at each of the three regional improvement bodies. At each of the six local test sites, one senior manager, one project manager and two frontline clinicians were interviewed (only one clinician was available for interview at two sites).

The interview schedule was structured around the core research questions and was developed iteratively by the four authors and then piloted with two programme participants. All interviews were conducted by the same researcher (EC). Interviews lasted on average around 1 hour and were digitally recorded and transcribed. These transcripts amounted to around 150 000 words.

### Data analysis

Given the focused nature of the research questions and the known structure of the Framework, thematic analysis was conducted using a template analysis approach to explore the key areas of activity and impact of the Framework and the programme. Template analysis is particularly helpful for analysing the perspectives of different groups within organisational settings.^19^ All data analyses were conducted in NVivo V.11 Pro. A coding template was constructed to analyse the interview data. This template was refined and expanded in the light of two pilot interviews to ensure that information and insights provided by participants were adequately captured by the template. The final coding template was then used to analyse all the interview transcripts (EC). A sample of the coded interviews (25%) was cross-checked by a second researcher (CM) and coding disparities were discussed, and adjustments made. The final coding was then reviewed by a third researcher not involved in data collection or template development but familiar with both the Framework and the programme (JC).

The final coding template was organised around five main themes. First, the organisational context and local work activities provided the background to the interview. Second, the role of the Framework concepts, including how these were understood, how they were disseminated and how useful and engaging these concepts were. Third, the clinical and organisational impact of the programme, including changes in thinking, working practice and data use. Fourth, the broader changes prompted by the programme. Fifth, the challenges and conditions required for wider use of the Framework, including the role of programme leaders and frontline individuals and teams. In practice, interviewees spoke most about the concepts and impact of the programme and this is reflected in the attention given to these topics in the findings. The findings are organised according to the themes that emerged from the analysis. We also provide a case example to illustrate the approach of one region that fully addressed all aspects of the Framework.

## Findings

The MMS Framework provided a useful, and sometimes challenging, way of thinking about safety and structuring measurement and monitoring strategies. Although some participants found it difficult to understand and apply the Framework in its entirety, a striking impact of using the Framework holistically was the broadening of the programme participants’ perspective on the components of safety. The tangible impacts of the Framework on organisational practice were, however, largely limited to the application of a small set of familiar safety interventions.

### Engaging with the Framework: thinking differently about safety

The most substantial reported impact of the Framework was the way in which individuals and teams within the programme thought about safety measurement and monitoring. There was agreement among all interviewees that the Framework changed the language people used, gave them a more holistic view of safety and encouraged them to reflect on how safety was actively managed in their environment.

#### Developing deeper understanding

It took time and effort for participants to understand fully the concepts underpinning the Framework. Participants differentiated understanding ‘the individual elements’ of the Framework (senior nurse, Org C) from grasping its purpose: ‘I think there’s a difference between understanding the Framework, in a sense of reading it and understanding what it means, and understanding how it actually applies’ (programme lead, Org D).

All interviewees agreed that that ‘it took a good six months […] to be confident and comfortable with the language of the report’ (safety manager, Org C) and to grasp the concepts behind the Framework and their implications in their local setting. The turning point was attributed to the role of external speakers (primarily authors of the report) who made sense of the concepts, for example, by putting a relevant patient story at the centre of the Framework or by going in depth into the concepts behind each domain.

#### Shifts in scope and scale

All interviewees, both clinical and managerial staff, described a change in their thinking as a result of participating in the programme, although the nature and depth of change varied considerably. Some sites described a generic improvement in ‘people’s day-to-day consciousness of safety’ (senior doctor, Org F), while other sites reported ‘a huge cultural shift; now we are talking about safety as opposed to managing risks’ (safety manager, Org A). This latter view was described as a shift away from a narrow focus on past incidents, risk assessments and performance management, to a deeper and more rigorous interrogation of what safety means that encompasses both system and ‘softer’ cultural factors, such as patient feedback and safety culture. This was expressed as a realisation that: ‘it’s about the *how* and *with whom* you have the conversations about the data, that’s where the value [of the Framework] is’ (safety manager, Org E).

#### A common language for safety

A near-universal theme in the interviews was that the Framework provided a common language for talking about safety measurement and monitoring: ‘the Framework has just become part of our language, we now all seem to be talking about the same thing and there’s less misunderstanding’ (project lead, Org E). Participants described noticing that everyone was ‘on the same page’, irrespective of their prior knowledge (programme manager, Org G). Several participants also described their perception that the Framework had encouraged a more open and non-threatening conversation about learning: “it has started different conversations about safety at all levels of the system. It is not a mandated, ‘this is the way that things should be,’ rather it is, ‘have you considered thinking about things this way?’” (improvement manager, Org H).

#### From assurance to inquiry

The underlying message of the Framework resonated strongly with many participants. This was described as moving from a space of compliance and assurance to one where safety is approached with a ‘more mindful and inquisitive’ mindset (safety manager, Org A). Managerial staff described realising the ‘powerful potential’ of the Framework ‘to fundamentally alter the types of questions we’ve been asking about our organisations, the sorts of data that we should be looking at, and the intelligence we should be drawing from it’ (improvement director, Org H). For frontline staff, this change in mindset towards safety measurement and monitoring took a different form. This was commonly characterised as a shift from seeing the collection of safety data ‘as just targets’ to recognising that ‘this information is beneficial to improving patient safety on the ward’ (programme manager, Org G).

### Clinical and organisational impact of the Framework

The practical impact of the Framework was highly variable across the provider organisations. Two test sites in particular (which were supported by the same regional body) approached the Framework in line with its intended purpose,^10^ and described using it as a lens to develop a more holistic approach to safety. Interviewees at these two sites regarded the Framework as the driver of much of these new ideas and initiatives.

Participants from the remaining sites indicated that they mainly focused on a few Framework domains, rather than using it holistically: ‘the three questions we used were: were we safe yesterday, are we safe today, and will we be safe in the future? […] virtually leaving out the reliability and the integration and learning domains’ (programme lead, Org A). These sites did achieve meaningful local level changes, although through focusing on testing a narrower range of safety interventions. Here the MMS Programme seemed to act more as a useful vehicle for local safety improvement, rather than a testing of a broader approach to safety measurement and monitoring.

#### A focus on single interventions

Staff were proud of achieving meaningful practical impacts, which largely took the form of single safety interventions, most notably safety huddles and briefings. For example, one site described how: ‘the safety huddle is a big thing that everyone’s talking about, even the cleaner and the ward pharmacists, because that’s a very visible physical change’ (project lead, Org B). These activities were described by some participants as reflecting the Framework emphasis on the critical role of the day-to-day monitoring of safety, but participants also reported that they were influenced by other ongoing regional improvement initiatives involving safety huddles. Other interventions (both in mental health settings) included an improved drug trolley round and creation of a comfort room to prevent aggression; staff described how these new ideas emerged as a direct result of their teams reflecting on the Framework.

#### Patient involvement with safety

The involvement of patients with safety measurement and monitoring and the collection of soft intelligence,^20^ as highlighted by the Framework, was an area of focus for all the test sites. Sites experimented with inquiring with patients about the state of the ward or running focus groups to ask about patients’ experiences of safety. In one mental health site, patients, staff and volunteers “co-designed a poster, ‘How we keep ourselves safe on the ward’” (project lead, Org E), which encouraged patients to become active in promoting their own safety.

In terms of safety monitoring, staff at all sites reported they had developed greater awareness of the value of patient experience as a barometer of safety. There was little indication by interviewees, however, that sites had been able to make the practical shift to investing time into developing routine usage of patient interviews and triangulating this with other safety information sources to identify patient safety risks.

#### Consolidating and integrating measures

The Framework was intended to provide an organising structure to bring together and understand a range of safety data. Examples of changes to usage of safety information that were made by sites during the programme included: the creation of a safety dashboard mapped to the five Framework domains with both daily and weekly monitoring, improved monitoring of existing metrics (eg, monitoring the incident reporting system) and implementation and monitoring of new metrics (eg, ‘number of ambulances off-road’).

Two sites reported that they extended the work beyond the frontline and made changes to their organisational-level reporting and interrogation of data. These more ambitious changes included: redesigning the clinical governance performance review, including testing a new set of specific measures and changing the format of governance and board meeting agendas and report templates to reflect the Framework domains.

Only one site reported they had stopped the collection of a redundant measure; for all other sites the lack of progress in this area was described as a real source of frustration: ‘it still feels like we haven’t got very far with changing our measures considering that this is a measurement programme’ (senior nurse, Org E). Staff felt there was considerable scope to abandon some routine reporting and replace it with more meaningful safety data, as encouraged by the Framework, but that the MMS Programme had not supported them sufficiently to achieve this goal.

### Broader changes prompted by the programme

Although the primary focus of the programme was testing the Framework in particular frontline care settings and services, test sites also saw some broader changes. At a regional level, two improvement bodies ran wider board education sessions which drew on Framework concepts; this work is still ongoing and has been extended to national level in one region (box 2).

One direct consequence of the programme’s series of regional and national learning events was the creation of a multidisciplinary network of like-minded clinicians and managers: ‘there’s something about bringing people together to talk about different experiences […] we’re learning more from each other’ (senior nurse, Org B). Teams went on site visits to the different trusts and used positive enquiry methods to share best practice. A cocreated e-guide captures much of this learning.^21^

Occasionally sites saw some unexpected broader changes. For example, one test site which was adversely affected by external pressures and internal instability reported few changes to their safety practices and processes. However, staff described how their participation in the programme ‘changed the conversation very significantly at a higher level’ (project lead, Org C), as evidenced by their board creating a new role for Head of Safety.

### Challenges and conditions for engaging with and using the Framework

The Framework was embraced and adopted with enthusiasm by some of the regional bodies and units, but others struggled to make effective use of it. Some participants described how the concepts and examples set out in the detailed Framework documentation were difficult to grasp, but it was clear that leaders who fully understood the concepts were able to communicate and translate the ideas for the local context.

#### The importance of leaders to explain and translate the concepts

The majority of the wider frontline staff across all the test sites relied on a summary ‘practical guide to the Framework’ and did not read the full report. Leaders in some regional and local sites developed a deep understanding of the Framework and were consequently able to communicate the core ideas to frontline staff who, in turn, could make use of the concepts and framework in their particular environment. As one interviewee reflected: ‘you need some stable leadership to enable change to occur, to empower staff to use the Framework in the right way’ (project lead, Org A). Other core programme participants said they had not read the full report; in these sites it was reported that the Framework was initially not well understood by many staff.

#### Unfamiliar concepts and approach

Participants who were experienced in safety and quality work clearly understood the value of an overarching conceptual model which drew on the wider safety literature and the experience of other industries. The unfamiliar concepts and technical language were more difficult for those with less experience. For example, a common perceived weakness of the Framework was the domain name ‘sensitivity to operations’, a term which many participants found off-putting as they associated it more with industry than healthcare.

A significant number of interviewees initially perceived the Framework not as a conceptual model but as a checklist or ‘a tool to directly influence specific aspects of safety’ (senior doctor, Org E). This misunderstanding of the nature and intended purpose of the Framework persisted for a few participants until the end of the programme.

### Practical challenges and conditions for change

Although the programme was well funded with support from dedicated regional and local programme leads, sites reported practical challenges in two key domains: skills and resources.

#### Baseline skills for engaging with the Framework

Participants described many reasons for the variability in the responses to the Framework. The most commonly cited reason was the variability across sites in the degree of existing maturity in safety and quality work, including capability in improvement and measurement, as well as broader knowledge of systems approaches to safety. Some sites were more confident that their teams were ready to engage with the Framework, whereas other sites realised they needed first to embark on more basic work. For example, one site ran human factors training at team ‘curry and learning evenings’ (project lead, Org B) which, while helpful, reduced the time they had available to engage teams with the Framework itself. Another participant described this need to ‘have a baseline level of understanding around safety and improvement’ as not ‘running before you can walk’ (project lead, Org I).

#### Essential resources for working with the Framework

Participants highlighted two fundamental criteria for working with the Framework on the frontline. First, enthusiastic frontline leaders were seen as instrumental in driving change and engaging teams through their capacity to ‘question things and not accept the norm’ (safety manager, Org C). The justification given was that the Framework ‘is not a ‘plug and go’ bit of kit; you’ve got to actually sit people down and engage them for it to have any meaning’ (safety manager, Org B).

Second, all participants described the importance of securing protected time for frontline teams to come together to reflect on new thinking around safety measurement and monitoring together. This reflective space was viewed as crucial for building a feeling of consensus and local ownership of the core principles, rather than being ‘done unto’ by a manager (safety lead, Org B).

### The potential of the Framework

All sites initiated valuable projects addressing at least one dimension of the Framework. However, only one region involved in the programme addressed all aspects of the Framework at both regional and frontline levels (box 2). Programme leaders in this region developed a thorough understanding of the core ideas, produced training materials for staff and made videos which brought the concepts to life, plus a variety of other activities and initiatives. They saw the importance of treating the Framework as a whole and consistently emphasised this in all their activities; they have built on this through a wider dissemination and implementation programme now under way across their health system.

Box 2 **Potential of the Measurement and Monitoring of Safety (MMS) Framework: a case study**

**National level application**

A healthcare improvement organisation sought to influence national patient safety programmes and policy through collaborating with public health bodies, stakeholder groups and the government. It also provided support to two frontline care organisations in their testing of the Framework:

**Test site 1: A multilevel application of the Framework**

The first organisation tested the Framework in an acute adult psychiatric ward. The team first mapped their current data and improvement activities to the Framework dimensions to identify gaps and conducted climate surveys to understand what safety meant to both their staff and their patients. This preparatory work led the team to select multidisciplinary team communication as their guiding theme. Nested within this, two work streams were identified: safety planning, which led to work with their board to develop a more specific set of measures for all mental health services, and medications safety, which produced specific interventions around reducing medication omissions on the ward, including completely changing the drug trolley round. Within each of these nested levels, the team applied the Framework to consider each aspect of the work holistically, as well as feeding their learning down, across and up the model as the programme developed.

**Test site 2: ‘Daily, weekly, monthly’ application of the Framework**

The second organisation tested the Framework using an innovative ‘daily, weekly, monthly’ approach across all levels of the organisation. The team implemented new daily ward and hospital-wide safety briefings with a script structured around the five Framework dimensions. On a weekly basis, visualisation of process, outcome and balancing measures through a new dashboard for safety measurement and monitoring aided managerial teams to make more informed decisions. The team also incorporated discussion of important qualitative safety information with quantitative measures using a structured format at each meeting. At monthly board-level meetings, report templates and agenda formats were revised to follow the Framework to structure conversations and encourage greater inquiry from decision-makers.

## Discussion

The MMS Programme aimed to assess the potential of a framework for safety measurement and monitoring^7^ as a means to stimulate a more productive and proactive approach to the measurement and monitoring of safety in healthcare organisations.

Using the MMS Framework provided a common language for safety, encouraged broader reflection and promoted a more holistic and proactive view of safety. Both frontline teams and managers used the Framework as a way of structuring their thinking about safety and as a prompt to asking questions about safety in their organisation. Programme participants talked about the difference between lagging and leading indicators of safety, of being more anticipatory, of seeing safety through the eyes of patients and of the importance of the monitoring element of safety: this constituted a substantial shift in mindset in both individuals and teams.

However, the changes in conceptual understanding did not always translate into changes in safety practices. Application of the Framework largely took the form of specific safety interventions (predominately huddles). These were valuable safety-related projects but taken overall they did not constitute much progress towards wider reform of the use and handling of safety information at the test sites. Several organisations primarily used the programme to give impetus and resource to existing initiatives.

It was suggested by those interviewed though that two frontline organisations had embraced the Framework and associated concepts in their entirety. These sites were distinguished by having committed leaders who had taken time to fully understand the concepts themselves, who maintained a flexible approach to exploring the utility of the Framework and who were willing to work with frontline staff to translate the concepts and apply them in local settings. The sites had considerable prior experience in safety and quality work and high-quality programme support from their regional improvement body which provided a solid foundation for the application of the Framework. This regional improvement body showed that a wider and deeper application of the Framework is possible at multiple levels of a health system (box 2). This region took time to engage fully with the concepts explained in the original report and to consider the examples of safety practices, adopting a cohesive approach throughout that used all five Framework dimensions.

The original Framework report was a commissioned research document and some sections (for instance on safety theories) were technical in nature. Despite the availability of practical examples in the report, extensive resources and testing of some effective communication strategies (eg, Framework champions, use of patient stories), some frontline staff still struggled to grasp the concepts. Programme participants who had not been given time to understand the underlying concepts understandably bypassed the conceptual understanding of the Framework to look for ‘the intervention’. A background in quality improvement among many (although not all) of the core programme staff, while conferring advantages in terms of existing capabilities and safety knowledge, seemed to influence how people approached the Framework.^22 23^

These mixed findings raise some useful considerations for other teams and organisations seeking to work with a conceptual model such as the Framework to change their thinking and behaviour around safety. Previous authors have set out useful models and requirements for the successful translation of evidence into practice and the implementation of improvement programmes.^24–26^ A focus on systems, ownership by local teams, support for technical work, local adaptation and a collaborative ethos are the essential underpinning for this work.^25^ However, some additional factors come into play with more conceptually based initiatives. First, any programme attempting to introduce unfamiliar concepts into frontline settings needs to build in adequate time to introduce such concepts. The MMS Programme was unusual, both in attempting to engender understanding of a new conceptual model and in seeking to test its practical application in diverse care settings. Second, programme leaders need to be well versed in the underlying ideas and to have sufficient expertise to translate them for frontline staff. Although the programme was well funded and supported, the concepts of the Framework were not initially conveyed accurately or in the depth required. As a result, misunderstandings about the concepts and purpose of the Framework persisted across many sites, in turn limiting the scope of the Framework application. Third, the nature of the ‘testing’ of a conceptual approach needs itself to be articulated and discussed. Confusion persisted for many participants as the programme tended to assume that sites understood what ‘testing the Framework’ meant, whereas in fact this is not a simple matter.

In hindsight it is clear that the MMS Programme required a more formal evaluation examining, at a minimum, changes in conceptual understanding, barriers and challenges, how the concepts were translated in frontline settings, potential impact on organisational priorities, changes to measurement and data management, and wider impacts. Disentangling the impact of the Framework itself from the generous funding and support of this programme could also be more explicitly addressed.

This study has some limitations which could be addressed in future programmes if evaluation is given a higher priority. The retrospective nature of this study did not allow for comparison against baseline data or enable full documentation of participants’ changes in thinking and practices over time. As such, insights gained from the post hoc interviews are limited by the extent to which participants could reflect on and recall their learning journey over the 18-month programme. We also accept that it was not ideal that the original authors of the Framework were so closely involved in the assessment of its impact in the first major programme of application. The interviewer (EC), however, had played no part in the original work and there was no indication that interviewees felt reluctant to voice criticisms of either the Framework or the programme. Future evaluations should of course be carried out by teams without a close association with the original report.

The Framework has now been applied in diverse settings and specialities, including paediatric intensive care, general practice and dentistry.^27–29^ The Canadian Patient Safety Institute and other international centres are currently drawing on the experience of the MMS Programme to develop their own safety measurement and monitoring programmes.^30^ The Canadian programme has drawn on the experience of the first use of the Framework in England and Scotland and particularly on the importance of preparatory workshops with Framework experts to ensure a solid understanding of basic concepts. In the UK, The Health Foundation has funded a second stage of the MMS Programme to develop additional learning and resources to support wider dissemination and application of the Framework.^21^ These programmes will allow a fuller assessment and evaluation of the potential of the Framework to promote a richer assessment of organisational safety.

## Concluding reflections

The testing conducted in the MMS Programme, combined with the wider testing and dissemination of the Framework in the UK and elsewhere, indicates the potential of the MMS Framework across healthcare specialties, settings and locations. Introducing new concepts within such programmes, however, requires committed leaders who themselves understand the concepts and more time to establish understanding and aims than might be needed in a standard improvement programme.

We found that the most powerful effects were in how people conceptualised safety and the need for active monitoring and anticipation as the fundamental feature of a safe organisation. We have come to describe the underlying shift in view as being one of moving from ‘assurance to inquiry’, meaning that while assurance processes can provide a bedrock of standards and reliable processes, safety is also highly reliant on constant questioning and active inquiry at all levels of an organisation. It is strange that the idea of an integrated surveillance system to measure and monitor safety remains so unusual in healthcare; in this respect, healthcare undoubtedly lags behind other high-risk industries.^8^ Healthcare organisations themselves can do much to improve integration and learning from safety and quality data but this could be greatly encouraged and stimulated by regulatory organisations. Rather than inspecting standards and processes, regulators might more productively ask organisations to ‘please demonstrate your safety measurement and monitoring system’ and ask how integration and learning is achieved at every level of the organisation.
